# Locally produced lactic acid bacteria for pathogen inactivation and odor control in fecal sludge

**DOI:** 10.1016/j.jclepro.2018.02.276

**Published:** 2018-05-20

**Authors:** Emmanuel Alepu Odey, Zifu Li, Xiaoqin Zhou, Yichang Yan

**Affiliations:** School of Energy and Environmental Engineering, Beijing Key Laboratory of Resource-Oriented Treatment of Industrial Pollutants, University of Science and Technology Beijing Xueyuan 30, Beijing 100083, PR China

**Keywords:** Fecal sludge, Coliform bacteria, Lactic acid bacteria, Fermented rice flour, Brown sugar

## Abstract

Providing safe fecal sludge (FS) sanitation has remained an important goal of global communities because of the high risks imposed on human health of the exposure to un-sanitized FS. This study used lactic acid fermentation as a pre-treatment technology to evaluate the sanitization effect of lactic acid bacteria (LAB) on FS. A combination of fermented rice flour and brown sugar was used as the medium to prepare LAB, and fecal coliforms were used as the indicator organisms. The addition of a LAB suspension grown in fermented rice flour and brown sugar to FS was studied to evaluate the survival of fecal coliforms. The pH decreased during ongoing lactic acid fermentation after the addition of the LAB suspension. The results revealed that fecal coliforms in reactors containing 1:1 and 2:1 w/w of FS and LAB suspension decreased to half of the initial concentration within seven days of the treatment process in comparison with that of the control reactor. Viable plate counts of 0.6 × 10^8^, 0.9 × 10^8^, and 2.4 × 10^8^ CFU/100 mL were recorded from reactors 1:1, 2:1, and the control, respectively. The total elimination of the fecal coliforms below the detection limit (<3 log 10 CFU/100 mL) was observed in both reactors after 15–17 days, whereas the number of fecal coliforms remained at 2.3 × 10^8^ CFU/100 mL in the control reactor. The fecal coliforms were eliminated because of the acidification caused by the LAB during the incubation time. The final pH in the treatment reactors 1:1 and 2:1 was 3.7 and 3.9. While the final pH in the control reactor was 7.91. The results revealed that the bacterial pathogens in FS can be completely eliminated through a low-cost technique and a simple lactic acid fermentation process.

## Highlights

• A combination of fermented rice flour and brown sugar was used to produce lactic acid.
• Fecal coliform was inactivated within a short period of time.
• Fecal pH was permanently reduced during treatment process.
• Fecal pathogen was eliminated through a low cost and easy to use lactic acid fermentation technique.

## Graphical abstract

**Figure:**
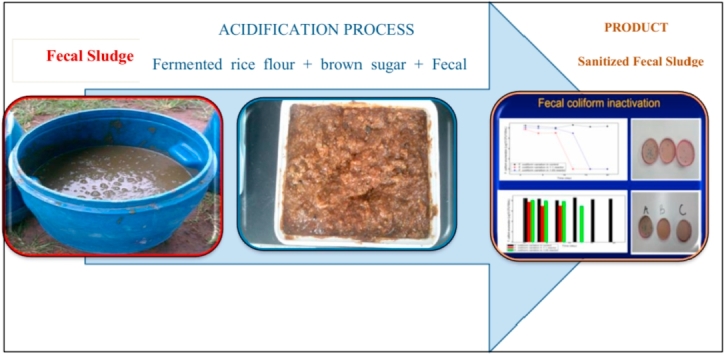

## 1 Introduction

Recent works aim to improve the sustainability of fecal sludge (FS) management for resource recovery (Andreev et al., 2018, Bracken et al., 2005; World Health Organization (WHO) & (UNICEF), 2014). Human feces are a natural fertilizer that can replace chemical or mineral fertilizers (Andreev et al., 2018, Factura et al., 2011, Glaser B and Guggenberger, 2001; Yemaneh et al., 2014). Given its nutrient contents, human feces could increase the soil fertility to achieve sustainable agriculture (Kimetu et al., 2004). However, the high concentrations of pathogens and several harmful organisms that are found in feces can contaminate the soil and crops (al-Ghazali and al-Azawi, 1990, Bracken et al., 2005, Jiang et al., 2002). Fresh human feces contains high amounts of pathogens, such as fecal coliforms, Helminth eggs, Escherichia coli, Salmonella spp, fecal streptococcus, and other bacteria, all of which are harmful to humans (Gold et al., 2017, Langergraber and Muellegger, 2005, Yemaneh, 2015). The direct use of FS in agriculture results in unhygienic conditions (Dold and Holland, 2011, Forbis-Stokes et al., 2016, Langergraber and Muellegger, 2005).

The different techniques typically used to reduce the infectious potential of feces vary in effectiveness. Several treatment methods, including ammonia treatment, lime addition, yeast treatment, and lactic acid fermentation (LAF), have been proposed to reduce the pathogens in FS (Anderson et al., 2015a, Factura et al., 2011, Feachem et al., 1983). Although these methods have been proven feasible, they are hindered by several drawbacks. For example, although lime treatment can increase the settling efficiency, the pH declines again after the initial reaction, thereby requiring the addition of high amounts of lime. In addition, bacteria pathogens can regrow over time. The concerns in this process include, lime scaling, ammonia odors and pathogen regrowth after a few days (Strande et al., 2014). In particular, ammonia treatment is applicable in areas with urine diverting dehydrating toilets (Nordin et al., 2009). The cost increases in cases where synthetic urea should be applied, thereby limiting the economic feasibility and sustainability of the technology (Strande et al., 2014). Another constraint is the stability of nitrogen in end-treatment products and the achievement of full-nutrient benefits. Ammonia disinfection is effective in urine, sewage sludge, and compost (Adamtey et al., 2009, Allievi et al., 1994); however, its applications to FS are still in the research phase of development (Magri et al., 2015).

The stabilization of FS through a fermentation and acidification process is one of the most reliable methods for pathogen inactivation and odor control (Mackie Jensen et al., 2008, Verbyla et al., 2016, Yemaneh et al., 2012). LAF is a cheap method that can be achieved through acidification at pH < 4 (Anderson et al., 2015b). LAF of feces is a simple and economical method that can be achieved through acidification (Andreev et al., 2017a). However, a number of LAB species do not produce effective lactic acids for pathogen inactivation in FS. For example, cassava is known for producing LABs such as *Pediococcus, Lactobacillus fermenttum*, and *Lactobacillus plantarum* (Colehour et al., 2014, Lacerda et al., 2005, Sanni et al., 2013). When lactic acid from fermented cassava flour was used to sanitize FS, the pH and pathogens did not significantly decrease (data not shown). Thus, effective and cheap lactic acid from food wastes must be produced to sanitize FS and preserve its nutrient value as fertilizer and reduces energy input.

Fermented rice water allows the growth of LAB (Li et al., 2015). However, no concrete information is available as regards the effectivity of the lactic acid produced from the combination of fermented rice flour and brown sugar for the inactivation of pathogens in FS. Given that the fermentation of rice flour is generally a non-controlled procedure, the LAB production through this process varies (Li et al., 2015, Strande et al., 2014). Therefore, to produce effective lactic acid from rice, the microbial growth and population during fermentation must be understood.

This study provided insight into the sensitive practicality of the resource-oriented sanitation of FS through lactic fermentation. The results of this study would benefit the FS management in developing countries and emergency situations, such as refugee camps, because the LAF of FS can eliminate pathogens and reduce unpleasant odors while improving its fertilizing value.

## 2 Materials and method

### 2.1 Lactic acid fermentation experimental set up

Two separate sets of experiments were conducted: one for obtaining lactic acid from the combination of fermented rice flour and brown sugar and another for investigating the sanitization effect of the lactic acid on FS under different treatment processes. The experiments were performed at room temperature (20 °C–25 °C). The experiment for obtaining lactic acid from fermented rice flour and brown sugar was performed on a single run, whereas that for investigating the sanitization effect of the lactic acid on FS was performed in triplicates.

For the lactic acid experiments, lactic acid was produced from fermented rice flour by adding brown sugar for a six-day fermentation process. Approximately 0.02 kg of brown sugar was added to 1.98 kg w/w of rice flour during the fermentation process. The main stages involved in producing lactic acid from fermented rice flour include boiling, cooling, and fermenting with the addition of brown sugar. Soaking is important for the production of fermented rice flour because it increases the water content of rice and enhances the natural fermentation process. 50 mL of deionized water was added to enhance the fermentation. The process was conducted in a wooden box and covered with a woolen material. Fermentation increased the lactic acid content of the process because of the metabolism of the involved LAB (Lu et al., 2005). The fermentation process in rice flour relies on the activities of LAB, such as Pediococcus spp., Lactobacillus, filamentous fungi, and yeasts.

0.1 mL of serially diluted samples were placed on LAB agar plates by using a piston-driven air displacement pipette and incubated at 37 °C for one day in an upside down position (Standard Method 9215). The LAB formed whitish colonies on the LAB agar plates. The pH level was monitored after 1, 5, 10, and every 24 h.

### 2.2 Origin of the fecal sludge

The FS used in the experiments was collected from the toilet septic tank of the University of Science and Technology Beijing. The characteristics of the FS are provided in Table 1. The FS was collected on the same day of the fermentation with the prepared lactic acid and transported to the laboratory in a 10 L bucket. In the laboratory, a suction pump was used to fill each of the three 5 L buckets with approximately 1 kg for the control bucket, 1 kg for the bucket marked for 1:1 of the FS and fermented rice flour and brown sugar, 1 kg for the third bucket marked for 2:1 of the FS and fermented rice flour and brown sugar. Prior to the fermentation experiments, 50 g of raw FS was collected for analysis of the physical and microbial properties.

**Table 1:** Initial fecal sludge characteristics.

		Initial fecal sludge characteristics
Parameters	Unit	Values
Temperature	°C	21
pH		7.53
Total solid (control)	%	14.41
Total solid (1:1 reactor)	%	35.6
Total solid (2:1 reactor)	%	21.1
Fecal coliforma	CFU/100 mL	3.1 × 10^8^

Fecal coliforms were used as indicator organisms to assess the overall sanitation efficiency of the locally made lactic acid. Evaluating the treatment efficiency of the FS requires measuring different kinds of pathogens, which is a labor-intensive and costly process. Thus, the typical alternative is to select and measure indicators of pathogenic activity to obtain an approximation of the level of pathogen removal during the treatment process (Strande et al., 2014). Coliform bacteria inhabit the intestinal tract and are widespread in FS. As such, their presence in the environment is used as an indicator of fecal contamination (Strande et al., 2014).

### 2.3 Treatment with optimal parameters

The lactic acid used in the experiment was produced from the optimal concentrations of fermented rice flour and brown sugar of 1.98 and 0.02 kg w/w, which amount to 2 kg. The first reactor contained 1 kg of FS and 1 kg of fermented rice flour and brown sugar (i.e., 1:1 w/w). The second reactor contained 1 kg of FS and 0.5 kg of fermented rice flour and brown sugar (i.e., 2:1 w/w). The third reactor contained 1 kg of FS and was used as the control. The experiment was conducted in triplicates. Samples were collected from all reactor containers daily for the pH test and every three to five days for the LAB and viable cell count of the lactic acid and the fecal coliform bacteria.

### 2.4 Odor evaluation

The odor strength of the FS during fermentation with rice flour was evaluated by six people. The potency of the perceived odor was evaluated by using a scale ranked from 0 (no odor) to 6 (very strong odor) as described in Table 2 (Andreev et al., 2017b, Misselbrook et al., 1993).

**Table 2:** Rank scale for different perceived fecal odor strengths.

Perceived odor strength	Rank scale
No odor	0
Very faint odor	1
Faint odor	2
Distinct odor	3
Strong odor	4
Very strong odor	5
Extremely strong odor	6

### 2.5 Analytical method

The FS sanitation experiment was monitored by measuring the concentration and accumulation of the lactic acid bacteria and the pH reduction. The ability of lactic acid to inactivate the fecal coliform was determined by evaluating the survival rates of the pathogens through a total viable plate count method and on the basis of the pH changes during the treatment process. The total solid content of the FS before and that after treatment was assessed by adopting the 2540E standard method for wastewater examination. The pH value during the fermentation of the lactic acid and the FS were determined by collecting a 10 g sample from each reactor, which was then dissolved in 100 mL distilled water. The dissolved portions were stirred for 15 min. After settling, the liquid portion was measured through potentiometric measurement with the standard pH electrode. The total viable counts of bacterial colonies on solidified agar plates that were obtained by chromocult coliform agar technique was used to measure the level of fecal coliform. Serial dilution of the fecal samples was performed in deionized water. The fecal coliform count was determined using membrane method with coliform agar, followed by incubation at 48 °C for 24 h. The treatment period was set as the time required for the fecal coliforms to appear on the agar. By using the Chromocult^®^ Coliform Agar, the number of fecal coliforms (CFU/100 mL) was determined for every analyzed sample. The culture media was prepared using standardized protocols and reagents under sterile conditions. This process was conducted in an electric oven operated aerobically.

## 3 Result and discussion

### 3.1 Lactic acid fermentation process

**Fig. 1:**
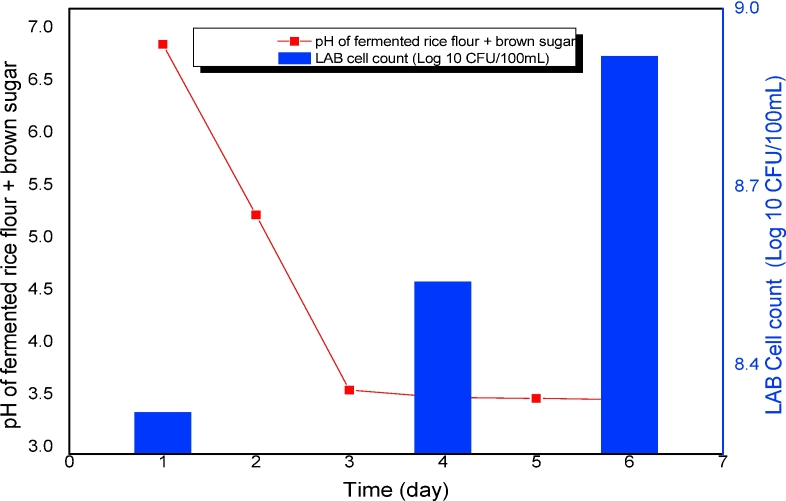
pH variation and LAB cell count during the fermentation of rice flour and brown sugar.

Fig. 1 shows the lactic acid fermentation process of rice flour and brown sugar. pH variation is one of the most important parameters that must be observed during lactic acid fermentation, because it reflects the acidification in the experiment (Kunene et al., 2000, Zhu et al., 2006). In this study, the pH of fermented rice flour and brown sugar reached below 4.0 after 48 h when brown sugar was added and remained at 3.51–3.62. This result was attributed to the rapid growth of LAB during the fermentation process. These LABs metabolized sugar to obtain energy and produced lactic acid as the end product. The increase in LAB concentration resulted in a pH drop.

This finding implied that fermented rice flour could be an effective lactic acid conditioner for pathogen inactivation in FS, because pathogens, such as *Staphylococcus aureus,* coliform, and *Salmonella* spp., rarely survive in acidic environments (Glaser B and Guggenberger, 2001). The LAB counts in the process increased by 8 log CFU/100 mL after 24 h. The increasing trend of the LAB concentration during the fermentation process could enable it to permanently suppress pathogens. Thus, fermented rice flour was used for the subsequent sanitization of the FS.

### 3.2 Lactic acid bacteria variation in fecal sludge

During the treatment period, the lactic acid concentration in the treatment reactors remained stable relative to the initial concentration of the LAB in fermented rice flour. The probable reason for this phenomenon is that the LAB metabolized the brown sugars added during the fermentation process, thereby producing lactic acid (Yemaneh et al., 2014). The concentration of lactic acid did not decrease during the overall treatment process. Fig. 2a shows the LAB cell count in the reactors over time, and Fig. 2b depicts the LAB cell count on the final day of the treatment process on the LAB agar. In the starting period of the mixture of the lactic acid with the FS, the concentration of LAB decreased in both the 1:1 and 2:1 reactors, whereas the concentration of the LAB in the portion of fermented rice flour and brown sugar was slightly higher. The fluctuations in the LAB concentrations in the 1:1 and 2:1 reactors may be attributed to the competition between the LAB and the coliforms for survival in the FS. However, on day 7 of the treatment process, the LAB in the fermented rice flour became lower than that in the treatment reactors. The overall stability in the variation of the LAB concentration in the treatment reactors and the fermented rice flour and brown sugar beginning from day 14 confirmed the effectiveness of fermented rice flour in producing lactic acid bacteria for FS sanitization.

**Fig. 2a:**
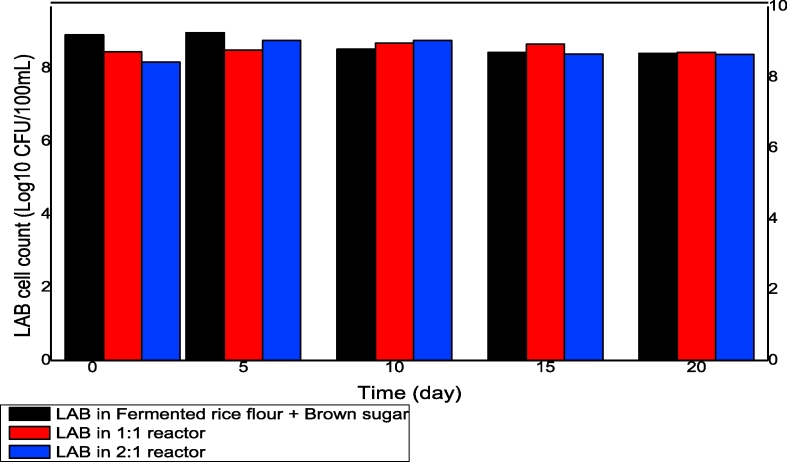
Lactic acid bacteria cell count in the reactors during the overall treatment process.

**Fig. 2b:**
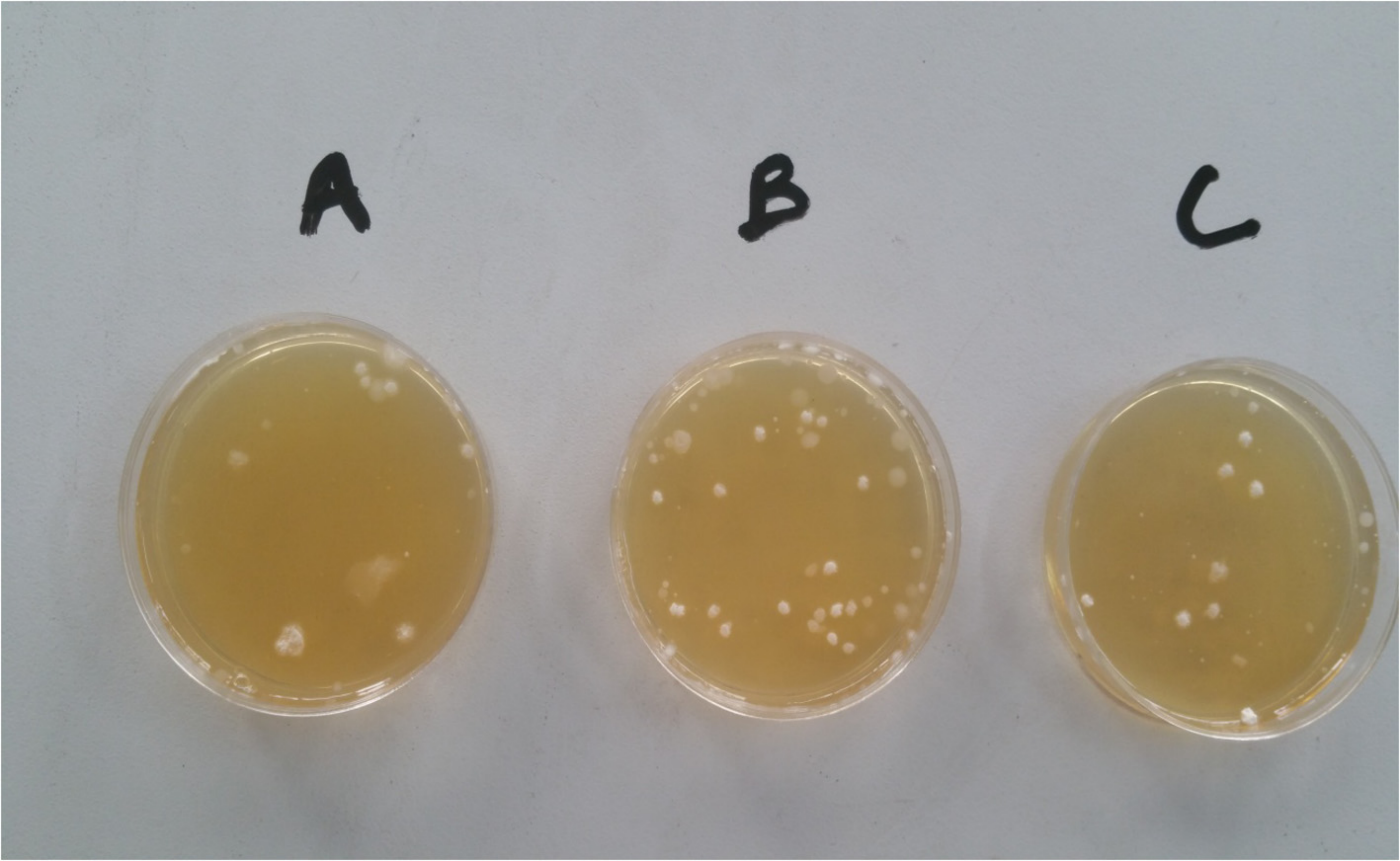
Lactic acid bacterial cell count on LAB agar plate 10^6^ (A = LAB in fermented rice flour, B = LAB in 1:1 reactor, C = LAB in 2:1 reactor).

### 3.3 Acidification of fecal sludge and pH reduction

**Fig. 3:**
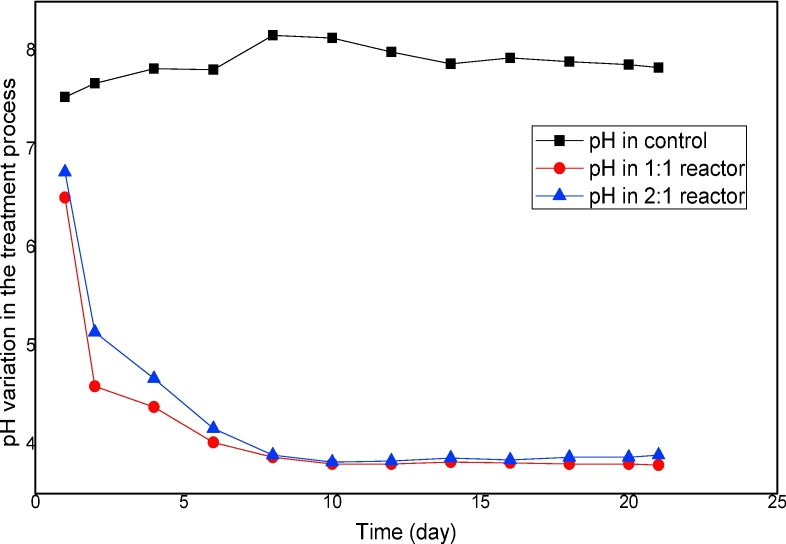
pH variation of fecal sludge during the treatment process.

Fig. 3 depicts the pH of the sanitization process. In the LAF experiments, the pH was monitored using two test reactors and a control reactor. The initial pH was 7.6 in the control reactor and 6.6–6.8 in the test reactors. The lower pH in the test reactors was due to the addition of lactic acid produced from rice flour and brown sugar, which has a pH of 3.5. For the reactor containing 1:1 of FS and fermented rice flour and brown sugar and that containing 2:1 of FS and fermented rice flour and brown sugar, the pH rapidly declined in the first seven days and remained nearly constant from day 10 to the final day of the experiment The increase in and stability of the LAB in the treatment process resulted in a rapid pH drop during the experiment. The reactor with 1:1 w/w mixture of FS with fermented rice flour and brown sugar had a final pH of 3.7, and that with 2:1 w/w had a final pH of 3.9. The pH in the control remained within 7.53–7.91 throughout the experiment.

These results suggested that two main factors influenced the sanitizing time and the efficiency of the lactic acid fermentation of FS, which include the combination of rice flour and brown sugar and the rapid drop of pH. According to Thimann (1963), lactic acid fermentation must have a final pH of 4.0 to be effective in sanitization. However, in addition to pH reduction, other antimicrobial compounds, such as hydrogen peroxide, bacteriocins, and diacetyl, are produced by LAB during the fermentation process, and these compounds induce an additional hygienization effect in the fermentation system. The final low pH recorded in this study was considered to have provided the desired hygienization and odor reduction effects. Observation of the pH profile in the LAF of the FS revealed that sugar addition is needed to reduce the pH such that an efficient LAF process can be achieved.

### 3.4 Fecal coliform inactivation

In assessing the efficiency of the treatment of FS, measuring different pathogens is a labor-intensive and costly strategy (Strande et al., 2014). A common practice to choose and measure indicators of pathogenic activity to approximate the amount of pathogens inactivated during the treatment process (Feachem et al., 1983, Strande et al., 2014). Fecal coliform was used in this experiment as the indicator organism to assess the effectiveness of fermented rice flour and brown sugar for pathogen inactivation in FS. Comparison of the results obtained for the treatment reactors and the control indicated that fecal coliform reduction was suppressed in the 1:1 and 2:1 reactors after seven days of fermentation, whereas the concentration of fecal coliforms remained high in the control reactor. The fecal coliform count reached below the detection limit (<3log10 CFU/100 mL) on day 15 in the 1:1 reactor and on the final day of fermentation in the 2:1 reactor, as shown in Fig. 4a, Fig. 4ba and b. In comparison, the samples collected from the control reactor showed only a slight reduction in fecal coliforms, as shown in Fig. 4b. The elimination of fecal coliforms during the treatment process was due to the pH decline in the process. Previous studies suggested that a pH range of 3.5–4.2 could efficiently eliminate various pathogens (Anderson et al., 2015c, Wang et al., 2001, Zhu et al., 2006). Gálvez et al. (2007) reported that a combination of different factors, such as organic acids, acidification, and antimicrobial compound production, enhances the lactic fermentation by LAB to eliminate undesirable microbes. The results of the present study suggested that the LAF process plays an important role in sanitizing and eliminating pathogens in FS.

**Fig. 4a:**
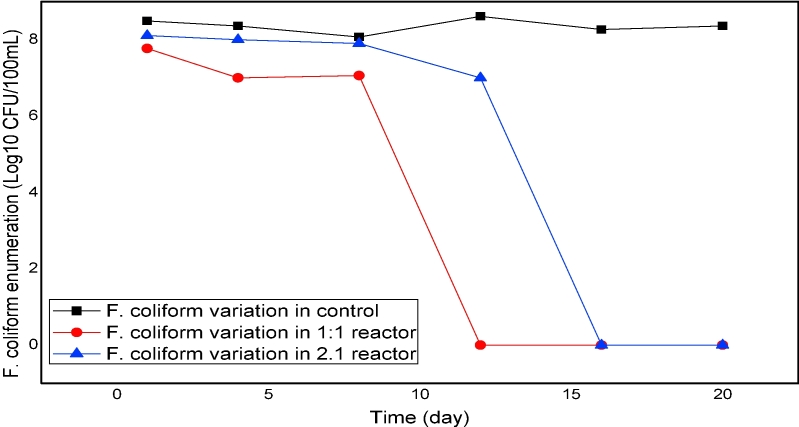
Fecal coliform variations during the treatment process.

**Fig. 4b:**
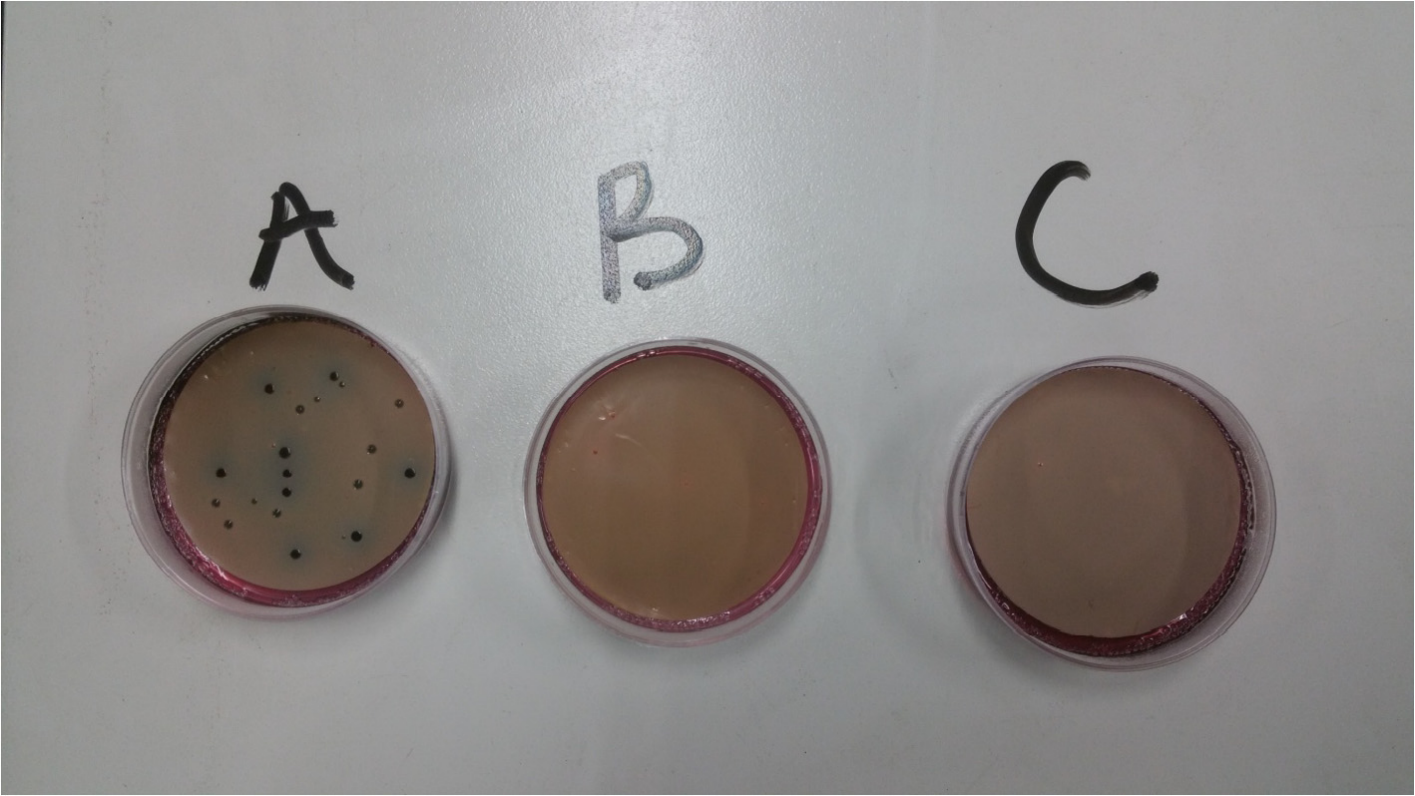
Fecal coliform cell count on the final day of treatment 10^6^ (A = control, B = 1:1 reactor, 2:1 reactor).

Fermentation reduced the pathogenic microorganism load in fecal waste in a more efficient and faster manner than composting. Liu (2016) found that although enterococci and coliform were reduced by composting for a period of months, traces of residual concentration remained in the mature compost. In view of the absence of a heating technique and the minimal gas production, this method can much more effectively conserve nutrients during the processing of FS.

### 3.5 Dewaterability of the treatment process

Table 3 presents the final characteristics of the FS from the three treatment reactors. The total solid (TS) was used as the metric of sludge dewaterability, and the results varied for each reactor. The TS of the control reactor was 13%, which was 1.41% lower than the initial TS. The % final TS values of the 1:1 and 2:1 reactors were 33.5% and 19.2%. Dewaterability significantly differed among the three reactors. The 1:1 reactor had higher TS than that of the control and 2:1 reactors. These results suggested that small changes in the dry matter content in the treatment reactors affected the fermentation process, and that FS dewaterability depended on the treatment process and thus cannot be predicted in any onsite sanitation facility (Gold et al., 2017, Yemaneh, 2015).

**Table 3:** Final fecal sludge characteristics from the treatment process.

Parameters	Unit	Final fecal sludge characteristics		
		Control	1:1 reactor	2:1 reactor
Temperature	°C	19	19	19
PH		7.9	3.7	3.8
Total solid (final)	%	13	33.5	19.2
Fecal coliform	CFU/100 mL	2.3 × 10^8^	0	0

### 3.6 Odor offensiveness of the treatment process

The qualitative responses from six observers to the questions regarding the presence of fecal odor and the acceptability of the perceived odor for agriculture use indicated that LAF process suppressed the odor from the FS and replaced it with a lactic acid smell in the 1:1 reactor, which was considered as a very faint odor by three observers; two observers reported a faint odor, and one observer reported a distinct odor. In the 2:1 reactor, the odor was replaced by a sour smell, which was considered a distinct odor by four observers and a faint odor by two observers according to (Misselbrook et al., 1993). The odor in the control reactor remained extremely offensive and was reported to be an extremely strong odor. Apart from the evaluation from the six observers, the variation of the odor was obvious to the researcher during the laboratory experiments and analyses. The effects of the LAF process on the removal of offensive odors from other organic wastes have been reported in other studies. Wang et al. (2001) reported that the odor was suppressed during the LAF processes of kitchen biowaste and fish waste. Huang et al. (2006) reported that the LAF of swine manure added with LAB reduced the odor. In the present study, the odor reduction could make the sanitized FS acceptable in agriculture as a soil amendment, because many users prefer odorless organic matter for soil amendment.

### 3.7 Efficiency analysis of LAF of FS

The results of this study demonstrated the potential feasibility of LAF technology application for FS sanitization using fermented rice flour and brown sugar. The technology was considerably effective in completely eliminating fecal coliforms within a short period of time, implying that the process was feasible and had a low reactor footprint and short retention time. Furthermore, the LAF technology had a relatively lower energy requirement than those of other conventional options, such as anaerobic digestion, microwave application, composting, liquid fuel, and dry fuel. Thus, in view of its potential for large-scale applications, this technology can be relevant in emergency situations, such as refugee camps, where the population and the FS generation are very high. The improvement of the food supply through agriculture by using sanitized FS as soil conditioner in emergency situations will be a welcome development. However, direct application of lacto-fermented feces to agriculture may be constrained by incomplete decomposition, high concentrations of organic acids or insufficient hygienization. Post-treatment by adding biochar, vermi-composting, or thermophilic composting stabilizes and sanitizes the material before its application for soil amendment.

This study validated the LAF application for the sanitization of FS under the tested method and laid the foundation for scaling up the method by assessing its energy consumption, process efficiency, and performance. When designing a large-scale LAF of FS technology for practical field application, food waste with LAF potential can be used to replace rice flour. One-third of the food produced globally for human consumption (approximately 1.3 billion tons) is lost. Developing and industrialized countries waste nearly the same amount of food (630 and 670 million tons annually, respectively) (News, 2011). Using part of these wastes for the LAF of FS will be an economic advantage.

## 4 Conclusion

In this study, FS treatment technology was evaluated using fresh FS collected from a toilet septic tank. This study demonstrated for the first time that the combination of fermented rice flour and brown sugar could be applied for the quick treatment of FS to reduce its pathogens and odor content before further treatment for soil amendment. The LAF of FS with fermented rice flour and brown sugar can be potentially applied as a low-cost and simple technique for effectively eliminating pathogens and odor emissions in FS. The addition of lactic acid produced from fermented rice flour and brown sugar led to an effective acidification of the process to pH of 3.51–3.52 and fecal coliform inactivation while maintaining high concentrations of LAB during the treatment process. The trends in the LAB composition in the FS during the treatment process revealed that LAB remained stable in the reactors, thereby enhancing the suppression of the fecal coliforms. From an economic perspective, this process could enable the storage of portions of the FS from the treatment reactor for a similar sanitization process, thereby reducing the need to purchase expensive LAB for the acidification process in the FS treatment. The results suggested that the FS treatment using fermented rice flour and brown sugar is feasible. Further investigations are needed to validate the use of food wastes to replace rice flour when considering large-scale applications and the efficiency of food waste as a LAF process for sanitizing other bacterial pathogens in FS.
